# Disparities in the Diagnosis and Treatment of Breast Cancer Among People With Disabilities

**DOI:** 10.1001/jamanetworkopen.2025.43559

**Published:** 2025-11-13

**Authors:** Hea Lim Choi, Jin-Hyung Jung, Hwa-Young Lee, Kyungdo Han, Dong Wook Shin

**Affiliations:** 1 Department of Clinical Research Design and Evaluation, The Samsung Advanced Institute for Health Sciences & Technology, Sungkyunkwan University, Seoul, South Korea; 2 Center for Trend Monitoring-Risk Modeling, Samsung Medical Center, Sungkyunkwan University School of Medicine, Seoul, South Korea; 3 Samsung Biomedical Research Institute, Sungkyunkwan University School of Medicine, Suwon, South Korea; 4 Graduate School of Public Health and Healthcare Management, The Catholic University of Korea, Seoul, South Korea; 5 Catholic Institute for Public Health and Healthcare Management, The Catholic University of Korea, Seoul, South Korea; 6 Department of Statistics and Actuarial Science, Soongsil University, Seoul, South Korea; 7 Department of Family Medicine/Supportive Care Center, Samsung Medical Center, Sungkyunkwan University School of Medicine, Seoul, South Korea

## Abstract

**Importance:**

Breast cancer is the most common cancer among women worldwide, yet individuals with disabilities may face substantial barriers affecting their diagnosis and treatment outcomes.

**Objective:**

To examine disparities in the diagnosis, treatment, and survival outcomes of patients with breast cancer and disability compared with those without disabilities.

**Design, Setting, and Participants:**

This retrospective cohort study linked the January 1, 2012, to December 31, 2019, Cancer Public Library Database records with the Korean National Disability Registration System, with follow-up through December 2020. The participants were women age 30 years or older with a diagnosis of breast cancer.

**Exposure:**

Presence and severity of medically verified disabilities, including physical, brain, sensory, and additional impairments.

**Main Outcomes and Measures:**

The primary outcomes were cancer stage at diagnosis, receipt of standard treatments (surgery, chemotherapy, or radiotherapy), and overall mortality. Multivariate logistic and Cox regression models adjusted for demographic, clinical, and socioeconomic factors. Statistical analyses were conducted in April 2025.

**Results:**

Among 150 412 women with breast cancer, 7443 (4.9%) had disabilities and 142 969 (95.1%) did not. Patients with disabilities were more likely to be aged 65 years or older (3068 patients with disabilities [41.2%] vs 20 182 patients without disabilities [14.1%]). In particular, those with severe disabilities were more likely than patients without disabilities to receive a diagnosis at distant (505 patients with disabilities [6.8%] vs 6601 patients without disabilities [4.6%]) or unknown (308 patients with disabilities [4.1%] vs 3518 patients without disabilities [2.5%]) stage. They were also less likely to undergo surgery (adjusted odds ratio [aOR] 0.91; 95% CI, 0.85-0.98), receive chemotherapy (aOR, 0.77; 95% CI, 0.73-0.81), or receive radiotherapy (aOR, 0.85; 95% CI, 0.79-0.90). The disparity was most pronounced for patients with severe brain impairments. Overall mortality was significantly higher among patients with disabilities (adjusted hazard ratio [aHR], 1.59; 95% CI, 1.50-1.69), particularly among those with severe disabilities (aHR, 2.15; 95% CI, 1.98-2.33).

**Conclusions and Relevance:**

In this cohort study of women with breast cancer, those with disabilities experienced significant disparities in stage at diagnosis, treatment access, and survival outcomes compared with those without disabilities. These findings highlight the critical need for targeted interventions to address disability-related barriers and ensure equitable health care.

## Key Points

**Question:**

Do patients with breast cancer and disability experience disparities in cancer diagnosis, treatment, and survival outcomes compared with those without disabilities?

**Findings:**

In this cohort study of 150 412 women with breast cancer in South Korea, 7443 patients who also had disabilities had significantly higher rates of advanced or unknown cancer stage at diagnosis, lower likelihood of receiving standard treatments, and higher overall mortality compared with those without disabilities.

**Meaning:**

These findings suggest that interventions addressing disability-related barriers are urgently needed to achieve equitable breast cancer care.

## Teaser

This cohort study examines disparities in the diagnosis, treatment, and survival outcomes of patients with breast cancer in Korea both with and without disability.

## Introduction

Breast cancer is the most commonly diagnosed cancer among women in South Korea and worldwide. In 2022, approximately 2.3 million women globally received a diagnosis of breast cancer, resulting in 670 000 deaths.^1^ Since 2019, breast cancer has been the predominant cancer among Korean women, accounting for 21.5% of new cases in 2021,^2^ and its incidence continues to rise.^3^

Addressing disparities in breast cancer diagnosis and treatment among disadvantaged populations, particularly those with disabilities, is crucial for improving overall cancer outcomes. Individuals with disabilities encounter barriers to timely and appropriate cancer diagnosis and care, resulting in poorer prognoses and higher mortality.^4^ These barriers include physical accessibility issues,^5,6^ biases among health care practitioners that affect clinical decisions,^4,7^ and socioeconomic disadvantages, such as lower income, education, or economic instability,^8^ which collectively hinder equitable access to timely screening, diagnosis, and treatment.

Existing studies have consistently reported worse outcomes in women with disabilities, such as lower screening rates,^9^ later-stage diagnoses,^10^ and reduced treatment access.^11^ In Japan and Taiwan, women with disabilities have lower screening participation,^12^ lower mammography uptake,^13^ higher odds of advanced-stage disease, and greater postdiagnosis mortality.^14^

However, existing studies have limitations. Most US studies define disability using the Medicare and Social Security Disability Insurance definition,^10,11^ which excludes working individuals and overlooks differences in insurance coverage. Age restrictions (eg, aged <65 years^11^ or >69 years^10^) further limit generalizability, and most studies lack chemotherapy data or consideration of disability severity. To date, no prior studies in Korea have examined survival after breast cancer by disability status.

In South Korea, a universal health insurance system covers all individuals, ensuring high accessibility to cancer care with minimal financial burden for cancer diagnosis and treatment. As of 2016, the copayment for cancer-related diagnostics and treatment was capped at just 5%, with a maximum out-of-pocket expense of approximately $1000 for low-income individuals.^15,16,17^ Additionally, Korea has implemented a national disability registration system that classifies types and severity of disabilities on the basis of standardized criteria and medical diagnoses.^18^ These structural features provide an ideal setting to analyze disparities in cancer care among individuals with disabilities.^19,20,21,22^ By utilizing Korea’s linked administrative database, our study investigates disparities in breast cancer diagnosis, treatment, and survival between individuals with and without disabilities.

## Methods

### Study Setting and Data Sources

#### Korean Clinical Data Utilization Network for Research Excellence

This cohort study utilized data from the Cancer Public Library Database (CPLD), established under the Korean Clinical Data Utilization Network for Research Excellence.^23,24^ The CPLD integrates cancer-related data from 4 population-based sources: the Korea National Cancer Incidence Database from the Korea Central Cancer Registry (eMethods in Supplement 1), cause of death data from Statistics Korea, healthcare utilization data from the National Health Information Database in the National Health Insurance Service, and the National Health Insurance Research Database in the Health Insurance Review & Assessment Service.^25^ These databases are linked using encrypted resident registration numbers, a 13-digit identifier assigned to all Korean citizens,^26,27^ enabling precise linkage while ensuring privacy and security.

The CPLD offers medical claims information, including diagnosis (*International Statistical Classification of Diseases and Related Health Problems, Tenth Revision *[*ICD-*10] codes), health care expenditure, inpatient and outpatient services, diagnostic tests, medical procedures, treatments, and prescription drugs.^28,29,30^ It contains demographic information such as age, sex, residential location, and income level, inferred from health insurance premiums, and data on general health check-ups and national cancer screenings. Due to its scope and detailed longitudinal information before and after cancer diagnoses, the CPLD is a crucial asset for research in cancer epidemiology and health policy.^31,32^ Prior studies have outlined the structure and accessibility of this database.^33^ Our study follows the Strengthening the Reporting of Observational Studies in Epidemiology (STROBE) reporting guideline for cohort studies. Approval for the study was granted by the institutional review board of Sungkyunkwan University. Informed consent was waived because the study utilized retrospective data.

#### Disability Registration System in Korea

This study utilized data on individuals with disabilities from the Korean Disability Registration System, a national registry allocating welfare benefits. This system classifies disabilities on the basis of medically verified conditions and functional impairments, categorizing individuals with disabilities into 15 types (ie, limb, brain, visual, auditory, linguistic, facial, kidney, heart, liver, respiratory, ostomy, epilepsy, intellectual, autism, and mental disabilities). Disability severity is ranked across 6 levels by medical specialists, on the basis of functional limitations and clinical impairment (eTable 1 in Supplement 1).^18,20^ In this study, disabilities were categorized as follows: (1) physical impairment (limb disability and facial disfigurement), (2) brain impairment (due to brain injury), (3) sensory impairment (visual and auditory disabilities), and (4) additional impairments, including internal organ impairment (heart, liver, kidney, respiratory diseases, epilepsy, or ostomy), mental impairment (intellectual, autism, or mental disorders), and linguistic disability. Disability severity was dichotomized into severe (grades 1-3) and mild (grades 4-6) categories.^19^

### Study Participants

The study population included all female individuals aged 30 years or older who received a diagnosis of breast cancer (*ICD-10* code C50) from January 1, 2012, to December 31, 2019 (158 243 individuals). Among these patients, only those with adenocarcinoma (153 476 individuals) were included. We excluded 3064 individuals with missing data for any covariates used in the analysis (eFigure 1 in Supplement 1).

### Statistical Analysis

Descriptive statistics were compiled according to the presence or absence of disabilities. For those with disabilities, further analyses were executed according to 5 predefined categories and severity levels. The χ^2^ test assessed differences in cancer stage by disability grades and types. Logistic regression analyses, adjusted for age, sex, Charlson Comorbidity Index (CCI; a weighted measure of comorbidity burden estimating mortality risk),^34,35^ income, residence, and cancer stage, estimated the likelihood of receiving specific treatments (surgery, radiotherapy, or chemotherapy). Income was categorized into deciles according to National Health Insurance premium amounts, with the first decile representing the lowest income group and the tenth the highest. Treatment was defined as initial therapy administered within 4 months after the first diagnosis. A series of analyses were conducted to investigate treatment disparities on the basis of disability presence, severity, and type, with patients with breast cancer without disabilities as the reference group.

To assess mortality risk, Cox proportional hazards regression analysis was employed to estimate hazard ratios (HRs) for overall and breast cancer–specific mortality among individuals with disabilities compared with those without disabilities. Survival time was tracked from the index date, the date of breast cancer diagnosis, to the earliest date of the following: death due to breast cancer, censorship (due to outmigration or other causes of death), or December 31, 2020, whichever came first. This analysis accounted for the treatments received, in addition to the covariables from the preceding treatment analyses. Survival probabilities were estimated using Kaplan-Meier curves, and differences according to disability status were evaluated via log-rank tests. We additionally analyzed mortality risk stratified by the Surveillance, Epidemiology, and End Results summary stage, and specifically examined the subgroup of patients with localized-stage breast cancer who underwent surgery, as this subgroup represented those with the highest potential for curative treatment and long-term survival. Analyses were conducted in April 2025 using SAS statistical software version 9.4 (SAS Institute). Two-sided *P* < .05 was considered statistically significant.

## Results

### Characteristics of Study Participants

Among the 150 412 patients with breast cancer, 7443 (4.9%) had disabilities and 142 969 (95.1%) did not. Those with disabilities were more likely to be aged 65 years or older (3068 patients [41.2%]) than those without disabilities (20 182 patients [14.1%]). Those with disabilities, compared with those without, exhibited a higher prevalence of comorbid conditions and a greater CCI score (mean [SD], 5.8 [2.9] vs 4.4 [2.5]). Individuals with disabilities were more frequently classified within the lowest income brackets (medical aid, first and second deciles) and were more likely to reside in nonurban areas than their counterparts without disabilities. Those with severe disabilities were older, had a greater prevalence of comorbidities, and belonged to the lowest income group more frequently. Among the disability types, those with brain impairments had the highest mean CCI score and prevalence of comorbidities and underwent surgery less often than did those with other disability types (Table 1).

**Table 1.** Baseline Characteristics of Patients With Breast Cancer^a^

Characteristics	Patients, No. (%)							
	Without disability (n = 142 969)	With disability (n = 7443)	Disability grade		Disability type			
			Mild (grade 4-6) (n = 4609)	Severe (grade 1-3) (n = 2834)	Physical (n = 3882)	Brain (n = 638)	Sensory (n = 1421)	Others (n = 1502)
Age group, y								
30-64	122 787 (85.9)	4375 (58.8)	2346 (50.9)	2029 (71.6)	2089 (53.8)	300 (47.0)	725 (51.0)	1261 (84.0)
≥65	20 182 (14.1)	3068 (41.2)	2263 (49.1)	805 (28.4)	1793 (46.2)	338 (53.0)	696 (49.0)	241 (16.1)
Charlson Comorbidity Index score								
Mean (SD)	4.4 (2.5)	5.8 (2.9)	5.7 (2.9)	5.9 (3.0)	5.6 (2.9)	6.8 (3.0)	5.5 (2.8)	6.1 (3.0)
2	40 705 (28.5)	1058 (14.2)	653 (14.2)	405 (14.3)	579 (14.9)	36 (5.6)	225 (15.8)	218 (14.5)
3	31 708 (22.2)	1074 (14.4)	699 (15.2)	375 (13.2)	607 (15.6)	62 (9.7)	233 (16.4)	172 (11.5)
4	16 713 (11.7)	885 (11.9)	549 (11.9)	336 (11.9)	480 (12.4)	59 (9.3)	197 (13.9)	149 (9.9)
5	53 843 (37.7)	4426 (59.5)	2708 (58.8)	1718 (60.6)	2216 (57.1)	481 (75.4)	766 (53.9)	963 (64.1)
Comorbidity								
Hypertension	35 275 (24.7)	3941 (53.0)	2552 (55.4)	1389 (49.0)	2037 (52.5)	460 (72.1)	779 (54.8)	665 (44.3)
Diabetes	12 475 (8.7)	1752 (23.5)	1103 (23.9)	649 (22.9)	871 (22.4)	190 (29.8)	338 (23.8)	353 (23.5)
Dyslipidemia	28 189 (19.7)	2792 (37.5)	1851 (40.2)	941 (33.2)	1478 (38.1)	301 (47.2)	527 (37.1)	486 (32.7)
Coronary heart disease	8991 (6.3)	1169 (15.7)	752 (16.3)	417 (14.7)	614 (15.8)	112 (17.6)	206 (14.5)	237 (15.8)
Stroke	671 (0.5)	139 (1.9)	73 (1.6)	66 (2.3)	51 (1.3)	49 (7.7)	21 (1.5)	18 (1.2)
Chronic obstructive pulmonary disease	18 192 (12.7)	1493 (20.1)	978 (21.2)	515 (18.2)	822 (21.2)	120 (18.8)	296 (20.8)	255 (17.0)
Income								
Medical aid, 1st-2nd decile	27 938 (19.5)	2664 (35.8)	1234 (26.8)	1430 (50.5)	1086 (28.0)	213 (33.4)	442 (31.1)	923 (61.5)
3rd-8th Decile	73 539 (51.4)	2971 (39.9)	2033 (44.1)	938 (33.1)	1721 (44.3)	237 (37.2)	614 (43.2)	399 (26.6)
9th-10th Decile	41 492 (29.0)	1808 (24.3)	1342 (29.1)	466 (16.4)	1075 (27.7)	188 (29.5)	365 (25.7)	180 (12.0)
Place of residence, urban	68 889 (48.2)	3344 (44.9)	2064 (44.8)	1280 (45.2)	1665 (42.9)	334 (52.4)	662 (46.6)	683 (45.5)
Treatment								
Surgery	117 587 (82.3)	5986 (80.4)	3814 (82.8)	2172 (76.6)	3212 (82.7)	452 (70.9)	1160 (81.6)	1162 (77.4)
Chemotherapy	75 964 (53.1)	3293 (44.2)	2014 (43.7)	1279 (45.1)	1690 (43.5)	233 (36.5)	652 (45.9)	718 (47.8)
Radiotherapy	29 356 (20.5)	1279 (17.2)	906 (19.7)	373 (13.2)	770 (19.8)	71 (11.1)	235 (16.5)	203 (13.5)
Hormone therapy	48 695 (34.1)	3036 (40.8)	1922 (41.7)	1114 (39.3)	1621 (41.8)	269 (42.2)	576 (40.5)	570 (338.0)

^a^ The percentages are based on the total number within each column group, except for the treatment variables, which are not mutually exclusive.

### Stages at Diagnosis According to Disability Status

The overall stage distribution at diagnosis was similar for patients with and without disabilities. However, individuals with disabilities were more likely than those without disabilities to receive a diagnosis at an advanced stage (383 [5.1%] patients with disabilities vs 6692 [4.7%] patients without disabilities) or to have an unknown stage at diagnosis (262 [3.5%] patients with disabilities vs 3617 [2.5%] patients without disabilities). This trend was particularly pronounced in patients with severe disabilities (179 [6.3%] patients with distant stage disease and 115 [4.1%] patients with unknown stage disease). The distant stage was more prevalent among patients with severe brain (25 patients [6.3%]) and sensory (26 patients [6.6%]) disabilities, whereas the unknown stage was more common across all types of disability types and severe grades, except for the other category, compared with patients with mild disabilities (Table 2).

**Table 2.** Adjusted Distribution of Stage at Breast Cancer Diagnosis by Disability Status, Severity, and Type^a^

Characteristic	Patients, No. (N = 150 412)	Patients by cancer stage, No. (%)^b^				*P* value^c^	*P* value^d^
		Localized (n = 88 608)	Locoregional (n = 50 872)	Distant (n = 7106)	Unknown (n = 3826)		
Disability status							
Without disabilities	142 969	84 475 (59.1)	48 185 (33.7)	6692 (4.7)	3617 (2.5)	<.001	NA
With disabilities	7443	4319 (58.0)	2479 (33.3)	383 (5.1)	262 (3.5)		<.001
Disability severity							
Severe (grade 1-3)	2834	1586 (56.0)	954 (33.7)	179 (6.3)	115 (4.1)	<.001	<.001
Mild (grade 4-6)	4609	2733 (59.3)	1525 (33.1)	204 (4.4)	147 (3.2)		.07
Disability type							
Physical	3882	2294 (59.1)	1293 (33.3)	167 (4.3)	128 (3.3)	<.001	.07
Brain	638	356 (55.8)	212 (33.2)	36 (5.6)	34 (5.3)		<.001
Sensory	1421	821 (57.8)	488 (34.3)	72 (5.1)	40 (2.8)		>.99
Others	1502	848 (56.5)	486 (32.4)	108 (7.2)	60 (4.0)		<.001
Disability type and severity							
Physical						<.001	
Severe	725	431 (59.4)	235 (32.4)	31 (4.3)	28 (3.9)		>.99
Mild	3157	1863 (59.0)	1058 (33.5)	136 (4.3)	100 (3.2)		.94
Brain							
Severe	398	212 (58.3)	139 (34.9)	25 (6.3)	22 (5.5)		.003
Mild	240	144 (60.0)	73 (30.4)	11 (4.6)	12 (5.0)		.69
Sensory							
Severe	394	218 (55.3)	136 (34.5)	26 (6.6)	14 (3.6)		>.99
Mild	1027	603 (58.7)	352 (34.3)	46 (4.5)	26 (2.6)		>.99
Others							
Severe	1317	726 (55.1)	443 (33.6)	97 (7.4)	51 (3.9)		<.001
Mild	185	122 (65.9)	43 (23.2)	11 (5.9)	9 (4.9)		.06

Abbreviation: NA, not applicable.

^a^ Adjusted for age, sex, income, place of residence, and Charlson Comorbidity Index score.

^b^ Percentages are calculated on the basis of the total number in each row.

^c^ Column shows overall *P* values by χ^2^ test for differences in stage distribution within each block.

^d^ Bonferroni-adjusted *P* values for pairwise comparison vs people without differences (reference) are shown.

### Association Between Disability and Cancer Treatment Received

Compared with patients without disabilities, those with disabilities were less likely to receive standard treatments, including surgery (5986 patients with disablity [80.4%] vs 117 587 [82.3%] patients without disability), chemotherapy (3293 [44.2%] patients with disability vs 75 964 [53.1%] patients without disability), and radiotherapy (1279 [17.2%] patients with disability vs 29 356 [20.5%] patients without disability) (Table 3). The adjusted odds ratios (aORs) were 0.91 (95% CI, 0.85-0.98) for surgery, 0.77 (95% CI, 0.73-0.81) for chemotherapy, and 0.85 (95% CI, 0.79-0.90) for radiotherapy. Disparities in treatment were more pronounced among individuals with severe disabilities, with aORs of 0.81 (95% CI, 0.73-0.90) for surgery, 0.66 (95% CI, 0.61-0.72) for chemotherapy, and 0.65 (95% CI, 0.58-0.73) for radiotherapy. Conversely, among individuals with mild disabilities, a significant disparity was noted only in chemotherapy treatment (aOR, 0.85; 95% CI, 0.79-0.90).

**Table 3.** Treatment Received by Disability Grades and Types

Characteristic	Patients, No. (N = 150 412)	Surgical intervention			Chemotherapy			Radiotherapy		
		Patients, No. (%) (n = 123 573 [82.2%])	OR (95% CI)		Patients, No. (%) (n = 79 257 [52.7%])	OR (95% CI)		Patients, No. (%) (n = 30 635 [20.4%])	OR (95% CI)	
			Crude	Adjusted^a^		Crude	Adjusted^a^		Crude	Adjusted^a^
Individuals without disabilities	142 969	117 587 (82.3)	1 [Reference]	1 [Reference]	75 964 (53.1)	1 [Reference]	1 [Reference]	29 356 (20.5)	1 [Reference]	1 [Reference]
Individuals with disabilities	7443	5986 (80.4)	0.89 (0.84-0.94)	0.91 (0.85-0.98)	3293 (44.2)	0.70 (0.67-0.74)	0.77 (0.73-0.81)	1279 (17.2)	0.80 (0.76-0.85)	0.85 (0.79-0.90)
Disability severity										
Severe (grade 1-3)	2834	2172 (76.6)	0.71 (0.65-0.77)	0.81 (0.73-0.90)	2014 (43.7)	0.69 (0.65-0.73)	0.66 (0.61-0.72)	373 (13.2)	0.59 (0.53-0.66)	0.65 (0.58-0.73)
Mild (grade 4-6)	4609	3814 (82.7)	1.04 (0.95-1.13)	0.99 (0.95-1.09)	4169 (45.5)	0.98 (0.93-1.02)	0.85 (0.79-0.90)	906 (19.7)	0.95 (0.88-1.04)	0.97 (0.90-1.05)
Disability type and severity										
Physical	3882	3212 (82.7)	1.04 (0.95-1.13)	1.00 (0.91-1.11)	1690 (43.5)	0.68 (0.64-0.73)	0.85 (0.79-0.91)	770 (19.8)	0.96 (0.91-1.08)	0.96 (0.91-1.07)
Severe	725	571 (78.8)	0.80 (0.67-0.96)	0.75 (0.61-0.92)	338 (46.6)	0.77 (0.67-0.89)	0.83 (0.71-0.97)	113 (15.6)	0.72 (0.58-0.87)	0.76 (0.62-0.93)
Mild	315	2641 (83.7)	1.11 (1.00-1.22)	1.05 (0.94-1.18)	1352 (42.8)	0.66 (0.62-0.71)	0.82 (0.76-0.89)	657 (20.8)	1.02 (0.93-1.11)	1.05 (0.96-1.14)
Brain	638	452 (70.9)	0.53 (0.44-0.62)	0.53 (0.43-0.65)	233 (36.5)	0.51 (0.43-0.60)	0.55 (0.46-0.66)	71 (11.1)	0.53 (0.41-0.68)	0.52 (0.41-0.67)
Severe	398	278 (69.9)	0.50 (0.40-0.62)	0.52 (0.40-0.67)	133 (33.4)	0.44 (0.36-0.55)	0.42 (0.33-0.53)	44 (11.1)	0.48 (0.35-0.66)	0.54 (0.39-0.74)
Mild	240	174 (72.5)	0.57 (0.43-0.76)	0.50 (0.36-0.70)	100 (41.7)	0.63 (0.49-0.82)	0.72 (0.54-0.96)	27 (11.3)	0.49 (0.33-0.73)	0.51 (0.34-0.77)
Sensory	1421	1160 (81.6)	0.96 (0.84-1.10)	0.96 (0.82-1.13)	652 (45.9)	0.74 (0.67-0.83)	0.93 (0.83-1.05)	235 (16.5)	0.77 (0.67-0.89)	0.78 (0.67-0.90)
Severe	394	311 (78.9)	0.81 (0.76-1.03)	0.86 (0.65-1.14)	181 (45.9)	0.75 (0.62-0.91)	0.83 (0.67-1.03)	53 (13.5)	0.60 (0.45-0.80)	0.63 (0.47-0.84)
Mild	1027	849 (82.7)	1.03 (0.88-1.21)	0.99 (0.82-1.19)	471 (45.9)	0.75 (0.66-0.85)	0.95 (0.83-1.09)	182 (17.7)	0.83 (0.71-0.98)	0.83 (0.70-0.98)
Others	1502	1162 (77.4)	0.74 (0.65-0.83)	1.00 (0.87-1.16)	718 (47.8)	0.81 (0.73-0.89)	0.69 (0.61-0.77)	203 (13.5)	0.69 (0.59-0.80)	0.68 (0.58-0.79)
Severe	1317	1012 (76.84)	0.72 (0.63-0.82)	0.97 (0.63-1.53)	627 (47.6)	0.80 (0.72-0.89)	0.62 (0.55-0.70)	163 (12.38)	0.55 (0.46-0.64)	0.63 (0.53-0.75)
Mild	185	150 (81.1)	0.93 (0.64-1.34)	0.98 (0.63-1.53)	91 (49.2)	0.85 (0.64-1.14)	0.84 (0.16-1.14)	40 (21.62)	1.07 (0.75-1.52)	1.10 (0.77-0.57)

Abbreviation: OR, odds ratio.

^a^ Adjusted for age, sex, Charlson Comorbidity Index score, income, place of residence, and cancer stage.

Patients with brain impairment presented the lowest likelihood of receiving any form of treatment, with aORs of 0.53 (95% CI, 0.43-0.65) for surgery, 0.42 (95% CI, 0.33-0.53) for chemotherapy, and 0.52 (95% CI, 0.41-0.67) for radiotherapy, across both mild and severe grades. Patients with physical disabilities also had significantly lower ORs for receiving treatments, but this was observed only in those with severe grades. Patients with sensory disabilities were significantly less likely to receive radiotherapy across both mild (aOR, 0.83; 95% CI, 0.70-0.98) and severe (aOR, 0.63; 95% CI, 1.47-0.84) grades (Table 3).

### All-Cause and Breast Cancer–Specific Mortality by Disability Status

Over a mean (SD) follow-up of 4.5 (2.3) years, 11 094 of 150 412 patients (7.4%) died. Patients with disabilities had a higher risk of overall mortality compared with those without (adjusted HR [aHR], 1.59; 95% CI, 1.50-1.69) and a lower 5-year overall survival rate (6028 patients [81.1%] vs 131 531 patients [92.4%]), (eFigure 2A and eTable 2 in Supplement 1). This disparity was more pronounced among individuals with severe disabilities (aHR, 2.15; 95% CI, 1.98-2.33), whose 5-year survival rate declined to 74.8% (2119 patients). By disability type, the greatest excess risk was observed in patients with brain impairments (aHR, 2.30; 95% CI, 1.92-2.75), who had the lowest 5-year survival rate (xx patients [69.7%]), followed by those with physical (aHR, 1.57; 95% CI, 1.31-1.90) and sensory (aHR, 1.40; 95% CI, 1.11-1.77) impairments. Breast cancer accounted for 8898 of all deaths (80.2%), and breast cancer–specific mortality patterns were similar to those of all-cause mortality (Table 4), with slightly higher survival rates (5-year survival, rates of 86.7% [6453 patients] and 93.6% [133 818 patients] for patients with and without disabilities, respectively) (eFigure 2B and eTable 3 in Supplement 1).

**Table 4.** Overall and Breast Cancer–Specific Mortality by Disability Grade and Type

Characteristics	Patients, No.	Overall mortality				Breast cancer–specific mortality			
		Deaths, No. (%)	Incidence rate/1000 PY	HR (95% CI)		Deaths, No. (%)	Incidence rate/1000 PY	HR (95% CI)	
				Crude	Adjusted^a^			Crude	Adjusted^a^
Individuals without disabilities	142 969	9820 (6.9)	51.3	1 [Reference]	1 [Reference]	8039 (5.6)	12.6	1 [Reference]	1 [Reference]
Individuals with disabilities	7443	1274 (17.1)	70.9	2.65 (2.50-2.80)	1.59 (1.50-1.69)	859 (11.5)	27.4	2.18 (2.03-2.34)	1.47 (1.36-1.58)
Disability severity									
Severe (grade 1-3)	2834	637 (22.5)	32.0	3.63 (3.35-3.93)	2.15 (1.98-2.33)	420 (14.8)	36.9	1.75 (1.59-1.93)	1.85 (1.67-2.04)
Mild (grade 4-6)	4609	637 (13.8)	56.0	2.08 (1.92-2.25)	1.26 (1.16-.1.37)	439 (9.5)	22.0	2.92 (2.64-3.22)	1.22 (1.11-1.35)
Disability type and severity									
Physical	3882	531 (13.7)	31.1	2.02 (1.85-2.21)	1.25 (1.14-1.37)	368 (9.5)	21.5	1.71 (1.54-1.90)	1.21 (1.09-1.35)
Severe	725	114 (15.7)	36.2	2.35 (1.96-2.83)	1.57 (1.31-1.90)	75 (10.3)	23.8	1.89 (1.51-2.37)	1.41 (1.12-1.76)
Mild	3157	417 (13.2)	29.9	1.95 (1.77-2.83)	1.18 (1.07-1.30)	293 (9.3)	21.0	1.67 (1.49-1.88)	1.17 (1.04-1.32)
Brain	638	181 (23.4)	73.0	4.73 (4.09-5.48)	2.20 (1.89-2.55)	112 (17.6)	45.1	3.57 (2.96-4.30)	1.95 (1.62-2.36)
Severe	398	125 (31.4)	83.17	5.39 (4.52-6.43)	2.30 (1.92-2.75)	80 (20.1)	53.2	4.20 (3.37-5.23)	2.12 (1.70-2.65)
Mild	240	56 (23.3)	57.25	3.71 (2.86-4.83)	2.00 (1.54-2.60)	32 (13.3)	32.7	2.59 (1.83-3.66)	1.63 (1.15-2.30)
Sensory	1421	206 (14.5)	35.6	2.31 (2.01-2.65)	1.33 (1.16-1.53)	142 (10.0)	24.5	1.94 (1.64-2.29)	1.31 (1.11-1.55)
Severe	1205	1063 (88.2)	743.1	2.92 (2.32-3.70)	1.40 (1.11-1.77)	51 (4.2)	32.4	2.56 (1.94-3.37)	1.38 (1.05-1.82)
Mild	444	357 (80.0)	593.6	2.08 (1.76-2.47)	1.30 (1.09-1.54)	91 (20.5)	21.6	1.71 (1.39-2.10)	1.27 (1.03-1.56)
Others	1502	356 (23.7)	60.0	3.89 (3.50-4.32)	1.33 (1.16-1.53)	237 (15.8)	39.9	3.15 (2.77-3.58)	1.31 (1.11-1.55)
Severe	1317	327 (24.8)	62.4	4.11 (3.68-4.59)	2.75 (2.50-3.10)	214 (16.2)	41.5	3.27 (2.86-3.75)	2.16 (1.88-2.48)
Mild	185	29 (15.7)	37.2	2.41 (1.69-3.47)	1.31 (0.91-1.89)	23 (12.4)	29.5	2.33 (1.55-3.51)	1.30 (0.86-1.96)

Abbreviations: HR, hazard ratio; PY, person-years.

^a^ Adjusted for age, sex, income, place of residence, Charlson Comorbidity Index score, cancer stage, surgery, chemotherapy, and radiotherapy.

### Survival According to Disability in Patients With Localized Disease Who Underwent Curative Surgery

Table 5 describes the results for patients with localized-stage disease who underwent surgery, while findings for patients with other stages are included in eTable 4 in Supplement 1. Patients with disabilities experienced significantly greater overall mortality than did those without disabilities (aHR, 2.41; 95% CI, 2.16-2.69). This disparity was particularly pronounced among those with severe disabilities (aHR, 4.14; 95% CI, 3.58-4.77) and less pronounced among those with mild disabilities (aHR, 1.63; 95% CI, 1.40-1.90). The highest mortality risk by disability type was observed in patients with brain impairments (aHR, 4.29; 95% CI, 2.26-5.49), followed by those with sensory (aHR, 1.92; 95% CI, 1.56-2.58) and physical (aHR, 1.75; 95% CI, 1.49-2.05) impairments. The overall mortality patterns were consistent with the breast cancer–specific mortality outcomes (Table 5).

**Table 5.** Overall and Breast Cancer Mortality by Disability Grades and Types in Patients With Localized-Stage Breast Cancer Who Underwent Surgery

Characteristic	Patients, No.	Overall mortality				Breast cancer–specific mortality			
		Deaths, No. (%)	Incidence rate/1000 PY	HR (95% CI)		Deaths, No. (%)	Incidence rate/1000 PY	HR (95% CI)	
				Crude	Adjusted^a^			Crude	Adjusted^a^
Individuals without disabilities	84 455	2147 (2.5)	5.6	1 [Reference]	1 [Reference]	1261 (1.5)	3.3	1 [Reference]	1 [Reference]
Individuals with disabilities	4153	393 (9.5)	21.3	3.83 (3.44-4.26)	2.41 (2.16-2.69)	166 (4.0)	9.0	2.75 (2.34-3.24)	1.95 (1.66-2.30)
Disability severity									
Severe (grade 1-3)	1466	209 (14.3)	33.4	6.02 (5.22-6.94)	4.14 (3.58-4.77)	86 (5.9)	13.8	4.21 (3.38-5.24)	3.16 (2.53-3.93)
Mild (grade 4-6)	2687	184 (6.8)	15.1	2.71 (2.33-3.15)	1.63 (1.40-1.90)	80 (3.0)	6.6	2.01 (1.60-2.52)	1.38 (1.10-1.73)
Disability type									
Physical	2258	165 (7.3)	15.8	2.84 (2.43-3.33)	1.75 (1.49-2.05)	72 (3.2)	6.9	2.12 (1.67-2.68)	1.47 (1.16-1.87)
Brain	325	66 (20.3)	47	8.47 (6.63-10.82)	4.29 (2.26-5.49)	31 (9.5)	22.1	6.77 (4.74-9.66)	4.08 (2.86-5.83)
Sensory	813	63 (7.7)	18.4	3.31 (2.58-4.26)	1.92 (1.56-2.58)	31 (3.8)	9.0	2.77 (1.94-3.96)	1.92 (1.34-2.74)
Others	757	99 (13.1)	30.9	5.56 (4.55-6.80)	4.47 (3.65-5.48)	32 (4.2)	10.0	3.05 (2.15-4.33)	2.55 (1.79-3.62)

Abbreviations: HR, hazard ratio; PY, person-years.

^a^ Adjusted for age, sex, income, place of residence, Charlson Comorbidity Index score, surgery, chemotherapy, and radiotherapy.

## Discussion

To our knowledge, this cohort study is the first nationwide, population-based study in Korea to comprehensively examine disparities in breast cancer care, including stage at diagnosis, treatment, and mortality, among individuals with disabilities. Previous studies have addressed incidence^36^ or screening^37^ disparities, but not these outcomes together. Strengths include a large, representative sample, inclusion of diverse disability types, and use verified disability diagnoses.

We found that the stage distribution at diagnosis was broadly similar between individuals with and without disabilities. However, late-stage diagnoses were more frequent in those with severe brain or sensory impairments, likely due to physical inaccessibility, communication barriers, and systemic health care bias. These findings align with previous studies suggesting that disability delays cancer detection by impeding access, communication, and screening.^38^ Women with brain-related disabilities were the least likely to participate in breast cancer screening.^37^

Patients with disabilities more often received diagnoses at unknown stages, reflecting incomplete staging assessments and inadequate treatment planning. Prior studies have shown that such patients undergo fewer recommended diagnostic evaluations,^11^ contributing to undocumented or undetermined cancer stages. Similar disparities have been reported in lung cancer.^19^ These gaps may stem from practitioner assumptions about limited prognosis or concerns about treatment tolerance, leading to suboptimal evaluations.^39^

We also found that individuals with disabilities were significantly less likely that those without disability to receive standard treatments, including surgery, chemotherapy, and radiotherapy, which is consistent with prior research.^11,40^ This disparity may result from an interplay of practitioner-related, patient-related, and system-level factors. Specifically, health care professionals may be less inclined to recommend aggressive cancer treatments for individuals with disabilities due to implicit biases regarding their quality of life, treatment tolerance, or expected prognosis.^41^ Studies have documented cases where physicians assume that patients with disabilities will have poorer treatment outcomes or lower functional recovery, leading to more conservative treatment recommendations.^39^ This phenomenon, often described as ableism in medicine, reflects societal attitudes that undervalue the lives and potential of individuals with disabilities, inadvertently influencing clinical decision making.^42^ Patients with disabilities themselves may forego treatment owing to personal, social, or logistical challenges. Some individuals may overestimate the risks of treatment-related complications or underestimate the benefits of therapy, leading them to decline potentially life-saving interventions. Practical barriers at the system level, such as transportation difficulties, mobility limitations, or the need for specialized support to attend daily radiotherapy sessions, may further limit access to appropriate care.^38^

Interestingly, our findings revealed that disparities in surgical treatment were less pronounced than those observed for chemotherapy or radiotherapy. This suggests that surgery may be a more accessible or tolerable treatment option for individuals with disabilities than other modalities, given that breast cancer surgery does not necessitate laparotomy or thoracotomy. Surgery is typically a 1-time intervention, whereas chemotherapy and radiotherapy require repeated visits, which may impose logistical and financial burdens on individuals with disabilities.

Treatment disparities were most pronounced among those with brain impairment. Notably, patients with severe brain impairment were significantly less likely to receive chemotherapy (aOR, 0.42). Brain impairment is often assessed via the modified Barthel Index, which is commonly used in patients with stroke^43,44^ (eTable 5 in Supplement 1). Individuals with severe brain injuries often face substantial mobility restrictions, including paralysis of both arms, making them dependent on full assistance for daily tasks. These physical limitations may pose substantial barriers to accessing chemotherapy, which requires multiple hospital visits. Physicians may also hesitate to recommend chemotherapy due to concerns about complications.^45^ Tailored decision-making tools and improved accessibility may help reduce treatment disparities in this vulnerable group.

Overall and cancer-specific mortality followed a similar pattern, and breast cancer was the primary cause of death (8898 [80.2%]). Breast cancer–specific mortality was significantly higher among individuals with disabilities (aHR, 1.47) and was even more pronounced among those with severe disabilities (aHR, 1.85). Even after adjusting for the treatments received, the mortality disparity persisted, suggesting that additional factors contribute to poorer outcomes in this population.

By disability type, breast cancer–specific mortality was highest in those with brain impairments (aHR, 2.12), followed by physical (aHR, 1.41) and sensory (aHR, 1.38) impairments. Contributing factors may include higher postoperative complications, unfavorable health behaviors,^46^ reduced treatment intensity,^45^ limited critical care access,^38^ and poor adherence to treatment.^47^ Additionally, adverse social determinants such as insufficient social support, inadequate caregiving assistance, and suboptimal living conditions among individuals with disabilities may further exacerbate health disparities.^48^ Importantly, the lack of significantly higher noncancer mortality underlines that individuals with disabilities should receive equal consideration for cancer treatment unless explicit contraindications are present.

Among patients with localized breast cancer who underwent surgery, mortality disparities remained substantial, particularly among those with severe disabilities. These results were adjusted for treatment factors, suggesting the inequities in postoperative care, follow-up adherence, and long-term cancer management may contribute to poorer outcomes in this surgically curable population.^4^ Interpretation of breast cancer mortality should also account for the possibility that death certificates may classify treatment-related complications as breast cancer deaths. While treatment disparities likely reflect systemic inequities,^4^ some differences may also represent appropriate clinical decisions for patients with increased vulnerability to treatment-related toxic effects. Cognitive impairments exhibited the highest mortality risk (aHR, 4.08), likely due to challenges in treatment comprehension, adherence, and postoperative care.^38,46,49^ Sensory and physical impairments were also linked to elevated mortality (aHR, 1.92 and 1.47, respectively), suggesting similar access and compliance issues.^50^ These findings highlight the need for a multidisciplinary approach to postoperative care, incorporating enhanced patient education, caregiver support, and rigorous monitoring of high-risk patients to ensure optimal treatment compliance and recovery.

### Limitations

This study has several limitations. First, data concerning the specific reasons why some patients did not undergo staging assessments or receive certain treatments were lacking. Second, we lacked detailed clinical data on breast cancer molecular subtypes, treatment intensity, patient adherence to postoperative and self-care protocols, or information on marital status and parity. Finally, our findings may not be fully applicable to other countries with different breast cancer epidemiology and health care systems.

## Conclusions

In conclusion, this cohort study found that patients with breast cancer and disability were less likely to receive comprehensive staging assessments and standard treatments compared with those without disabilities. Overall mortality was significantly higher among individuals with disabilities, with markedly greater disparities noted in those with severe and brain-related impairments. Most excess mortality was attributed to breast cancer, highlighting that unequal access to standard care substantially contributes to survival disparities. Our study reveals clear disability-related barriers among patients with breast cancer and underscores the need for targeted interventions to enhance accessibility, increase practitioner awareness, and ensure equitable cancer care.

## Notes

Supplement 1. eMethods. The Korea Central Cancer Registry (KCCR) eReferences eTable 1. Specialty Physicians Authorized to Issue Medical Certificates for Diagnosis in Various Disability Areas eTable 2. Overall Survival Rate in Post-Breast Cancer Diagnosis Among Women With Disabilities and Without Disabilities eTable 3.Breast Cancer-Specific Survival Rate in Post-Breast Cancer Diagnosis Among Women With Disabilities and Without Disabilities eTable 4. Overall and Breast Cancer-Specific Mortality by Disability Grades and Types in Patients at Regional, Distant, and Unknown Stage eTable 5. Definitions of Disability Grades Following Brain Injury eFigure 1. Flow Diagram of the Study Population Selection eFigure 2. Kaplan-Meier Curves Illustrating Survival Rates Among Breast Cancer Patients With And Without Disabilities

Supplement 2. Data Sharing Statement
