# Supplementary material for: Disparities in the Diagnosis and Treatment of Breast Cancer Among People With Disabilities
Source: JAMA Netw Open. 2025 Nov 13;8(11):e2543559. doi: 10.1001/jamanetworkopen.2025.43559 (PMC12616457; doi:10.1001/jamanetworkopen.2025.43559)
Supplement: Supplement 1. — eMethods. The Korea Central Cancer Registry (KCCR) eReferences eTable 1. Specialty Physicians Authorized to Issue Medical Certificates for Diagnosis in Various Disability Areas eTable 2. Overall Survival Rate in Post-Breast Cancer Diagnosis Among Women With Disabilities and Without Disabilities eTable 3.Breast Cancer-Specific Survival Rate in Post-Breast Cancer Diagnosis Among Women With Disabilities and Without Disabilities eTable 4. Overall and Breast Cancer-Specific Mortality by Disability Grades and Types in Patients at Regional, Distant, and Unknown Stage eTable 5. Definitions of Disability Grades Following Brain Injury eFigure 1. Flow Diagram of the Study Population Selection eFigure 2. Kaplan-Meier Curves Illustrating Survival Rates Among Breast Cancer Patients With And Without Disabilities [file jamanetwopen-e2543559-s001.pdf]

Supplemental Online Content

Choi HL, Jung J, Lee H, Han K, Shin DW. Disparities in the diagnosis and treatment of breast cancer among people with disabilities. *JAMA Netw Open.* 2025;8(11):e2543559. doi:10.1001/jamanetworkopen.2025.43559

Supplement 1. eMethods. The Korea Central Cancer Registry (KCCR)

eReferences

eTable 1. Specialty Physicians Authorized to Issue Medical Certificates for Diagnosis in Various Disability Areas

eTable 2. Overall Survival Rate in Post-Breast Cancer Diagnosis Among Women With Disabilities and Without Disabilities

eTable 3. Breast Cancer-Specific Survival Rate in Post-Breast Cancer Diagnosis Among Women With Disabilities and Without Disabilities

eTable 4. Overall and Breast Cancer-Specific Mortality by Disability Grades and Types in Patients at Regional, Distant, and Unknown Stage

eTable 5. Definitions of Disability Grades Following Brain Injury

eFigure 1. Flow Diagram of the Study Population Selection

eFigure 2. Kaplan-Meier Curves Illustrating Survival Rates Among Breast Cancer Patients With And Without Disabilities.

This supplemental material has been provided by the authors to give readers additional information about their work.

© 2025 Choi HL et al. *JAMA Network Open.*

eMethods

The Korea Central Cancer Registry (KCCR) is a nationwide cancer registry that has maintained comprehensive coverage of the Korean population since 1999**.** It collects crucial cancer-related information including patient demographics (age and sex), date of diagnosis, cancer site, and SEER summary staging (localized, regional, distant, or unknown)**.** Detailed descriptions of KCCR and KNCI DB have been provided in previous studies (Kang MJ et al., 2023; Shin HR et al., 2005), and their use has been validated by the method proposed by Ajiki et al.

eReferences

Kang MJ, Jung KW, Bang SH, et al. Cancer Statistics in Korea: Incidence, Mortality, Survival, and Prevalence in 2020. *Cancer Res Treat.* 2023;55(2):385-399.

Shin HR, Won YJ, Jung KW, et al. Nationwide cancer incidence in Korea, 1999~2001; first result using the national cancer incidence database. *Cancer Res Treat.* 2005;37(6):325-331.

Ajiki W, Tsukuma H, Oshima A. [Index for evaluating completeness of registration in population-based cancer registries and estimation of registration rate at the Osaka Cancer Registry between 1966 and 1992 using this index]. *Nihon Koshu Eisei Zasshi.* 1998;45(10):1011-1017.

© 2025 Choi HL et al. *JAMA Network Open.*

eTable 1. Specialty physicians authorized to issue medical certificates for diagnosis in various disability areas

	Specialists qualified to provide disability diagnoses (must be board-certified in relevant specialties)
Physical disability	Rehabilitation medicine physician, Orthopedic surgeon, Neurosurgeon, Neurologist, Rheumatologist
Visual disability	Ophthalmologist
Hearing disability	Otorhinolaryngologist
Speech and language disability	Otorhinolaryngologist, Psychiatrist, Neurologist, Maxillofacial surgeon
Intellectual disability	Psychiatrist, Neurologist, Rehabilitation medicine physician
Disability from brain injury	Rehabilitation medicine physician, Neurosurgeon, Neurologist
Disability associated with autism	Pediatric psychiatrist
Disability due to mental disorders	Psychiatrist
Disability due to renal failure	Internal medicine physician, General surgeon (Transplant)
Disability due to cardiac issues	Cardiologist, Pediatrician, Thoracic surgeon
Disability due to respiratory disorders	Pulmonologist, Allergologist
Disability due to liver disorders	Hepatologist, General surgeon (hepatobiliary), Pediatrician
Disability due to facial disfigurements	Plastic surgeon, Dermatologists, General surgeon (burn specialty)
Disability due to ostomies	General surgeon, Gynecologist, Urologist, Internal medicine physician
Disability due to epilepsy	Neurologist, Neurosurgeon, Psychiatrist, Pediatrician, Pediatric neurologists

© 2025 Choi HL et al. *JAMA Network Open*.

eTable 2. Overall survival rate in post-breast cancer diagnosis among women with disabilities and without disabilities

	Overall survival rate							
			by disability severity		by disability types			
Year after diagnosis	Without disability (n=142,969)	With disability (n=7,443)	Mild (n=4,609)	Severe (n=2,834)	Physical (n=3,882)	Brain (n=638)	Sensory (n=1,421)	Others (n=1,502)
1	0.987	0.956	0.967	0.939	0.964	0.929	0.968	0.937
2	0.971	0.915	0.935	0.882	0.933	0.856	0.930	0.879
3	0.953	0.875	0.901	0.833	0.903	0.795	0.891	0.822
4	0.937	0.843	0.87	0.792	0.881	0.752	0.858	0.771
5	0.924	0.811	0.85	0.748	0.851	0.697	0.828	0.740
6	0.910	0.787	0.827	0.723	0.830	0.659	0.810	0.710
7	0.899	0.767	0.811	0.695	0.814	0.607	0.797	0.682
8	0.889	0.740	0.787	0.662	0.798	0.552	0.774	0.629
9	0.878	0.712	0.758	0.631	0.767	0.531	0.729	0.620

© 2025 Choi HL et al. *JAMA Network Open*.

eTable 3. Breast cancer-specific survival rate in post-breast cancer diagnosis among women with disabilities and without disabilities

	Breast cancer-specific survival rate							
			by disability severity		by disability types			
Year after diagnosis	Without disability (n=142,969)	With disability (n=7,443)	Mild (n=4,09)	Severe (n=2,834)	Physical (n=3,882)	Brain (n=638)	Sensory (n=1,421)	Others (n=1,502)
1	0.990	0.967	0.974	0.956	0.973	0.942	0.973	0.959
2	0.975	0.936	0.950	0.913	0.949	0.886	0.947	0.913
3	0.960	0.905	0.923	0.875	0.926	0.837	0.914	0.870
4	0.947	0.884	0.906	0.846	0.909	0.826	0.896	0.829
5	0.936	0.867	0.891	0.827	0.890	0.820	0.880	0.813
6	0.926	0.855	0.878	0.816	0.880	0.804	0.866	0.799
7	0.918	0.847	0.873	0.806	0.875	0.753	0.863	0.797
8	0.910	0.832	0.862	0.781	0.867	0.729	0.857	0.752
9	0.903	0.824	0.849	0.781	0.854	0.729	0.848	0.752

© 2025 Choi HL et al. *JAMA Network Open*.

eTable 4. Overall and Breast Cancer-Specific Mortality by Disability Grades and Types in Patients at Regional, Distant, and Unknown Stage.

		Overall mortality				Breast cancer mortality			
	N	Number of deaths No. (%)	Incidence rate (per 1000 PY)	Crude HR (95% CI)	Adjusted HR (95% CI)	Number of deaths No. (%)	Incidence rate (per 1000 PY)	Crude HR (95% CI)	Adjusted HR (95% CI)
**Regional**									
Individuals without disabilities	48,395	3,809 (7.9)	17.6	Reference	Reference	3,185	14.7	Reference	Reference
Individuals with disabilities	2.477	393 (18.1)	42.7	2.44 (2.21-2.69)	1.60 (1.45-1.77)	304	29.0	1.98 (1.76-2.22)	1.47 (1.31-1.66)
By disability severity									
Severe (Grade 1-3)	992	213 (21.5)	52.0	2.97 (2.59-3.41)	2.14 (1.87-2.47)	139	34.0	2.32 (1.95-2.75)	1.84 (1.55-2.18)
Mild (Grade 4-6)	1,485	235 (15.8)	36.8	2.10 (1.84-2.39)	1.30 (1.14-1.48)	165	25.8	1.76 (1.51-2.06)	1.26 (1.08-1.48)
By disability types									
Physical	1,259	193 (15.3)	35.0	2.00 (1.73-2.31)	1.26 (1.09-1.46)	146	26.5	2.81 (1.53-2.13)	1.31 (1.11-1.55)
Brain	217	52 (24.0)	61.6	3.52 (2.68-4.63)	2.00 (1.51-2.62)	24	28.4	1.93 (1.30-2.88)	1.28 (0.86-1.91)
Sensory	473	72 (15.2)	36.1	2.07 (1.64-2.61)	1.28 (1.01-1.62)	46	23.1	1.58 (1.18-2.11)	1.14 (0.85-1.52)
Others	528	131 (24.8)	61.4	3.51 (2.95-4.18)	2.91 (2.44-3.47)	88	41.2	2.81 (2.27-3.47)	2.46 (1.99-3.05)
**Distant**									
Individuals without disabilities	6,601	3,356 (50.8)	163.1	Reference	Reference	3,227	156.8	Reference	Reference
Individuals with disabilities	505	331 (65.5)	267.7	1.67 (1.50-1.87)	1.14 (1.02-1.28)	316	255.5	1.66 (1.48-1.87)	1.26 (1.12-1.42)
By disability severity									
Severe (Grade 1-3)	246	170 (69.1)	294.2	1.85 (1.58-2.15)	1.39 (1.19-1.63)	162	280.4	1.83 (1.56-2.14)	1.51 (1.29-1.73)
Mild (Grade 4-6)	259	161 (62.2)	244.4	1.52 (1.30-1.79)	0.96 (0.81-1.12)	154	233.86	1.52 (1.56-2.14)	1.08 (0.91-1.27)
By disability types									

© 2025 Choi HL et al. *JAMA Network Open*.

Physical	208	127 (61.1))	227.6	1.41 (1.19-1.69)	0.91 (0.76-1.08)	121	216.8	1.40 (1.17-1.68)	1.01 (0.84-1.21)
Brain	56	39 (69.6)	347.2	2.22 (1.62-3.05)	1.28 (0.93-1.75)	38	338.3	2.25 (1.63-3.10)	1.54 (1.12-2.13)
Sensory	86	57 (66.3)	275.7	1.72 (1.33-2.24)	1.05 (0.81-1.36)	54	261.2	1.70 (1.30-2.22)	1.21 (0.92-1.59)
Others	155	108 (69.7)	359.5	1.89 (1.56-2.28)	1.65 (1.36-1.99)	103	286.5	1.87 (1.53-2.27)	1.67 (1.37-2.03)
**Unknown**									
Individuals without disabilities	3,518	508 (14.4)	34.2	Reference	Reference	366	24.6	Reference	Reference
Individuals with disabilities	308	102 (33.1)	91.2	2.69 (2.17-2.22)	1.45 (1.17-1.80)	73	65.3	2.67 (2.07-3.43)	1.66 (1.29-2.13)
By disability severity									
Severe (Grade 1-3)	130	45 (30.6)	97.2	2.57 (1.95-3.38)	1.30 (0.99-1.72)	33	71.3	2.90 (3.38-5.24)	1.87 (1.31-2.67)
Mild (Grade 4-6)	178	57 (32.0)	87.0	2.86 (2.11-3.88)	1.68 (1.24-2.28)	40	61.1	2.50 (1.81-3.47)	1.52 (1.09-2.11)
By disability types									
Physical	157	46 (29.3)	76,6	2.25 (1.67-3.05)	1.24 (0.91-1.67)	29	48.3	1.97 (1.35-2.88)	1.27 (0.87-1.86)
Brain	40	24 (60.0)	200.2	5.96 (3.96-8.98)	2.24 (1.49-3.37)	19	158.5	6.53 (4.12-10.4)	2.97 (1.87-4.72)
Sensory	49	14 (28.6)	87.2	2.57 (1.51-4.37)	1.12 (0.66-1.91)	11	68.5	2.78 (1.53-5.07)	1.46 (0.80-2.66)
Others	62	18 (29.0)	76.0	2.24 (1.40-3.58)	1.79 (1.12-2.86)	14	59.1	2.43 (1.43-4.13)	1.89 (1.11-3.22)

Abbreviations: HR, hazard ratio; CI, confidence interval; PY, person-years.

Adjusted for age, sex, income, place of residence, Charlson comorbidity score, surgery, chemotherapy, and radiotherapy.

© 2025 Choi HL et al. *JAMA Network Open*.

eTable 5. Definitions of disability grades following brain injury

Grade	Definition
1	Unable to walk independently; complete assistance required<br><br>Unable to perform any ordinary activities due to complete paralysis of both arms; complete assistance required<br><br>Unable to perform any ordinary activities due to complete paralysis of one arm and one leg; complete assistance required<br><br>Modified Barthel Index ≤32 points, necessitating complete assistance to perform all ordinary activities, including ambulation
2	Unable to perform any ordinary activities due to complete paralysis of one arm; complete assistance required<br><br>Unable to use all fingers of both hands due to complete paralysis and joint contracture; complete assistance required<br><br>Modified Barthel Index 33–53 points; complete assistance needed to perform most ordinary activities, including ambulation
3	Unable to use all fingers of one hand due to complete paralysis and joint contracture; complete assistance required<br><br>Unable to walk due to complete paralysis of one leg; significant assistance required<br><br>Modified Barthel Index 54–69 points, unable to perform ordinary activities independently; partial assistance required
4	Modified Barthel Index 70–80 points, capable of performing ordinary activities but requires intermittent assistance
5	Modified Barthel Index 81–89 points, able to perform most ordinary activities independently but occasionally requires assistance
6	Modified Barthel Index 81–89 points, able to perform most ordinary activities independently but occasionally requires extra time

© 2025 Choi HL et al. *JAMA Network Open*.

eFigure 1. Flow diagram of the study population selection.

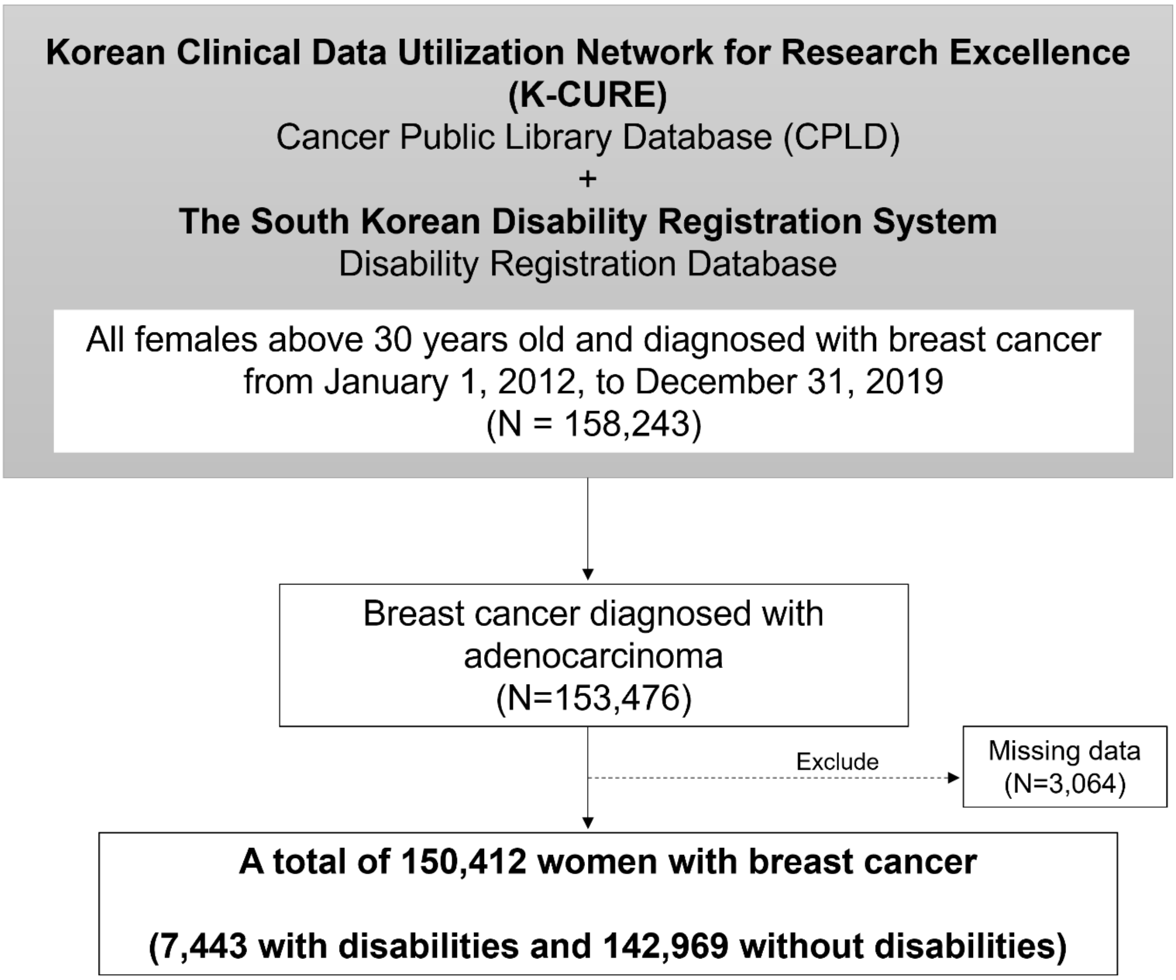

© 2025 Choi HL et al. *JAMA Network Open*.

eFigure 2. Kaplan-Meier curves illustrating survival rates among breast cancer patients with and without disabilities.

A. Overall survival in breast cancer patients with and without disabilities

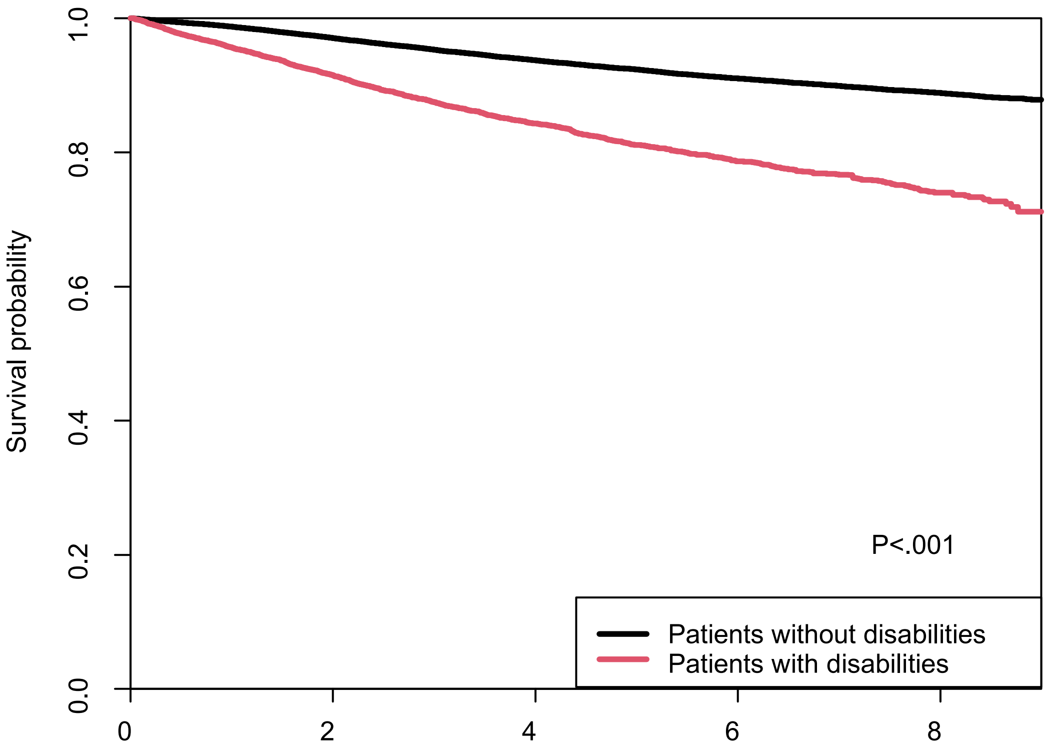

B. Breast cancer-specific survival in breast cancer patients with and without disabilities

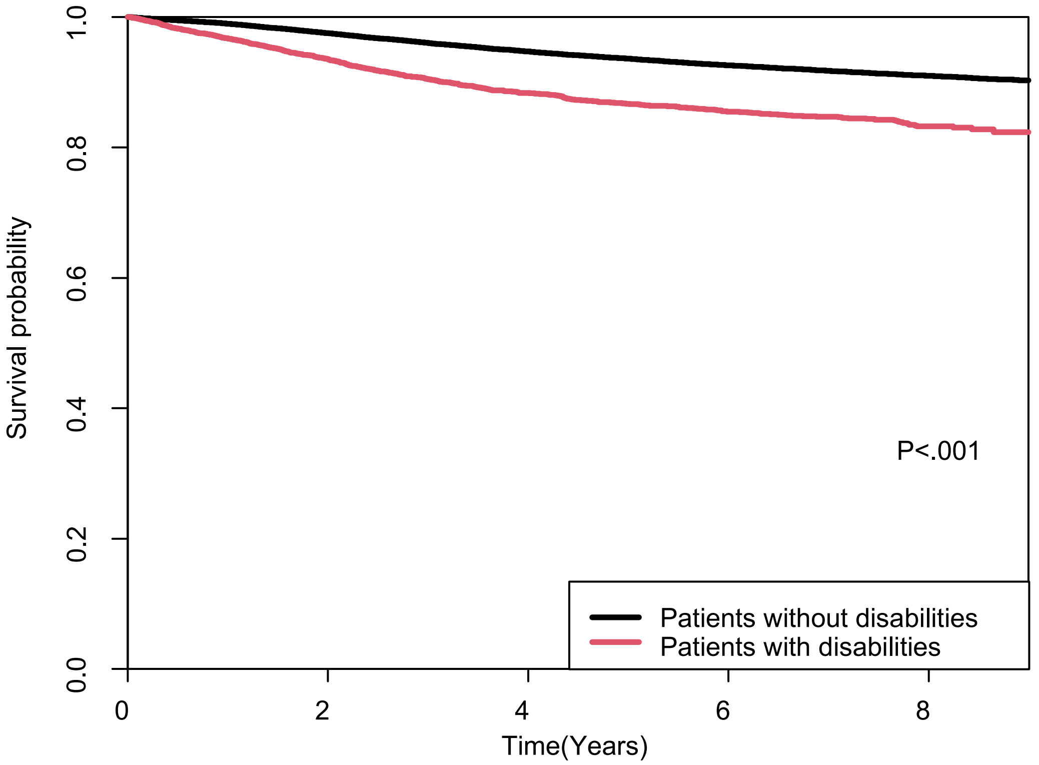

© 2025 Choi HL et al. *JAMA Network Open*.
