# Supplementary material for: Disparities in the Diagnosis and Treatment of Breast Cancer Among People With Disabilities
Source: JAMA Netw Open. 2025 Nov 13;8(11):e2543559. doi: 10.1001/jamanetworkopen.2025.43559 (PMC12616457; doi:10.1001/jamanetworkopen.2025.43559)
Supplement: Supplement 2. — Data Sharing Statement [file jamanetwopen-e2543559-s002.pdf]

Data Sharing Statement

Choi. Disparities in the Diagnosis and Treatment of Breast Cancer Among People With Disabilities. *JAMA Netw Open*. Published November 13, 2025.
doi:10.1001/jamanetworkopen.2025.43559

Data
Data available: No
